# An empirical study on the determinants of health care expenses in emerging economies

**DOI:** 10.1186/s12913-020-05414-z

**Published:** 2020-08-24

**Authors:** Lulin Zhou, Sabina Ampon-Wireko, Henry Asante Antwi, Xinglong Xu, Muhammad Salman, Maxwell Opuni Antwi, Tordzro Mary Norvienyo Afua

**Affiliations:** School of Management, Jiangsu University, Zhenjiang, 212013 P. R. China

**Keywords:** Health care expenditure, Industrialization, Agricultural activities, Economic growth, Quantile regression

## Abstract

**Background:**

Emerging countries continue to suffer gravely from insufficient healthcare funding, which adversely affects access to quality healthcare and ultimately the health status of citizens. By using panel data from the World Development Indicators, the study examined the determinants of health care expenditure among twenty-two (22) emerging countries from the year 2000 to 2018.

**Methods:**

The study employed cross-section dependence and homogeneity tests to confirm cross-sectional dependence and to deal with homogeneity issues. The Quantile regression technique is employed to test for the relationship between private and public health care expenses and its determinants. The Pooled mean group causality test is used to examine the causal connections among the variables.

**Results:**

The outcome of the quantile regression test revealed that economic growth and aging population could induce healthcare costs in emerging countries. However, the impact of industrialization, agricultural activities, and technological advancement on health expenses are found to be noticeably heterogeneous at the various quantile levels. Unidirectional causality was found between industrialization and public health expenses; whereas two-way causal influence was reveled amongst public health expenditure and GDP per capita; public health expenditure and agricultural activities.

**Conclusion:**

It is therefore suggested that effective and integrated strategies should be considered by industries and agricultural sectors to help reduce preventable diseases that will ultimately reduce healthcare costs among the emerging countries.

## Background

As a nation becomes wealthy, individuals begin to place more value on life and this results in demand for health services along with an inevitable escalation of health care spending. The tremendous increased elderly population requires intensive health care and the affluent tend to be more cautious about their health [1]. Recent estimate [2] suggest that global health spending could perhaps rise from US$8 trillion in 2018 to $18 trillion in 2040 with a projection of about 9% of the global GDP to be allocated to health 2040, according to the Institute for Health Metrics and Evaluation (IHME).^1^

Universal health coverage embodies national health systems in which all individuals can access quality health services without individual or familial financial hardship. Efforts to ensure universal access should include financial sustainability to increase public health expenses, whiles exploring options to broaden revenue sources through agricultural activities and industrialization and prioritizing the appropriate use of resources. In the midst of coronavirus pandemic, one of the major concerns is the need for additional resources to support the healthcare system. This calls for an urgent need to better understand the role of economic activities such as industrialization and agricultural activities on health care costs as a number of the population become infected within the selected emerging nations.

Although the theory of health spending could differ among nations, Poullier, Hernandez [3] offered a categorization of total health expenditure (THE). Total health expenditure is measured as a sum of private and public expenses of health care services. The public spending disbursements are generally financed by social security assistance, several forms of taxation from governments, and external sources in addition to loans and grants. The private expenses include out of pocket (OOP) payments and other private health services. According to the World Bank, almost 400 million people are deprived of access to essential healthcare services^4^. Evidence suggests almost 100 million people are hard-pressed into extreme poverty each year due to catastrophic health expenditure.^2^ The emerging countries are home to almost half of the global population. Yet the combined value of health expenditure is just USD 1.3trn, less than half of the US expenditure.^3^ The big gap in healthcare spending per capita between these countries and the remaining countries studied regarding the drivers of health care expenditure in recent research work can be described as scanty.

**Fig. 1:**
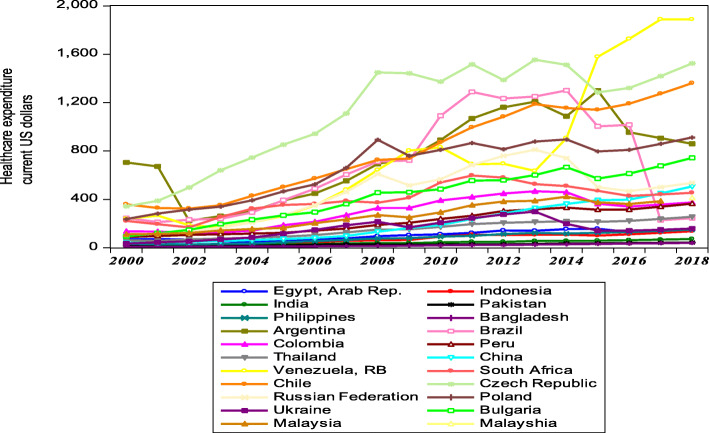
Public healthcare expenditure among Emerging economies from 2000 to 2018

Figure 1 shows the trend of public expenses on health care among the twenty-two (22) countries studied spanning from 2000 to 2018. It can be deduced that public health expenditure among the emerging nations has experienced a growing trend since the year 2000. Comparing the 22 countries, Venezuela became the country with the highest public healthcare expenditure from 2015 onwards which could be attributed to many factors including either to increase health care efficiency or to curtail certain outbreaks like diphtheria. Hence this increase in health expenditure may not always correspond to better health performance and this is supported by similar findings from [4]. Brazil experienced a decline in public health spending in 2018 whereas Bangladesh, however, remained the poor performer among the twenty (22) emerging nations to contribute the least of its public funds to support healthcare.

As these nations endeavor to move toward advanced healthcare systems, they unceasingly experience double disease burden^4^: on one hand, emerging countries are struggling to subside the incidence of infectious diseases and also meeting the demand of primary health care; on the other hand, they are at the same time faced with increasing cases of non-communicable diseases^5^ which is driven by unhealthy lifestyles and an escalating aging population.^6^ The health cost effect is compelling many governments to harness available financial capital more efficiently and also, prioritize the delivery of selective health services.

Over the past decades, reviewed multicity literature paid attention to identifying forces behind the growth of health expenditure. In investigating the factors influencing health care spending and the causes of increased health care expenditure in Malaysia, annual data covering the period 1981 to 2014 [4] using ordinary least square (OLS) and Autoregressive Distributed Lag Model (ARDL), it was clarified that technological progressions contribute to increasing health care spending in Malaysia. Murthy and Okunade [5] peruse the contributing factors of health expenses using the autoregressive distributed lag (ARDL) approach, using 1960–2012 annual time-series data and found medical technology been a major element driving increasing health care expenses in the United States. Han, Cho [6] researched factors determining health expenses among sixteen (16) provinces in Korea from the year 2003–2010 and found no significant interplay between income and health spending. In 2017, empirical findings of a study conducted by Kraipornsak [7] in Asia and the OECD countries found income to enhance health expenses. Ke, Saksena and Holly [8] examined the role of income, demographic factors and government revenues on total health spending. Di [9] assessed the effects of income, age distribution and healthcare spending. Thompson, Wells and Coasts [10] addressed the health needs of the vulnerable group. While the findings of these studies provide a useful guide to the understanding of significant variations in health care expenditure across countries, most of them relied on time series data, and ordinary least square, however to some extent the results and conclusions were inconsistent. With such limitation in the existence of heavy-tailed distributions and outliers, quantile regression usage is appropriate and applied in [11] to yield a more robust and accurate finding.

The study theoretically, provides a fresh look for understanding the relationship between economic activities and health expenditure. It is noteworthy to realize that most literature [12–15] focused on the developed nations regarding the effects of economic activities on health to the neglect of emerging nations. As questions and concerns about the role economic activities on health expenditure grow louder, understanding the role of industrialization and agricultural activities on public health spending in emerging nations become increasingly vital. Against this and as part of introducing innovation, the study pinned on the connections between industrialization and agricultural activities on total health expenditure in emerging countries, which in the view of the researchers is given little attention in the literature. The introduction of new variables and the use of recent data in the estimation make a new image of the traditional studies to provide support about how health management and policymakers can include new insights on the allocation of resources for health.

Besides, some studies have utilized other factors such as technological advancement, age, literacy rate to determine the aggregate form of total health expenditure in developing and developed nations [16, 17]. Hence ignoring the distinctive influence of such factors on private and public health expenditure in studies could lead to a lack of opportunity to propose specific policies. This study, therefore, disaggregated total health expenditure into public hand private health expenditure. The practical implication of this study is to help determine the magnitude of the explanatory variables on each of the two components of total health expenditure. This study is important, as there is a global interest in controlling the growth of expenditure on health and enhancing financial risk protection for all population groups [18]. The study will provide appropriate policy guidelines to achieve the sustainable development goal that aimed at increasing health financing specifically in developing nations.

In addition, a review of the recent literature on the factors influencing health expenses with panel data analysis utilized the first-generation econometric technique. The first-generation econometric technique governs by the assumption of cross-sectional independence and homogeneity within the panel data sets. However, relying on such assumption of cross-sectional independence and homogeneity could lead to spurious estimation results in cases where the results are rather cross-sectional dependent and heterogeneous. The study, therefore, employed the second-generation technique, which considers the existence of the issue of cross-sectional independence and homogeneity to enable the selection of appropriate estimation procedures. Using a new approach for panel data, quantile regression appears to be more promising in providing robust results to fill the gap in the empirical literature.

**Fig. 2:**
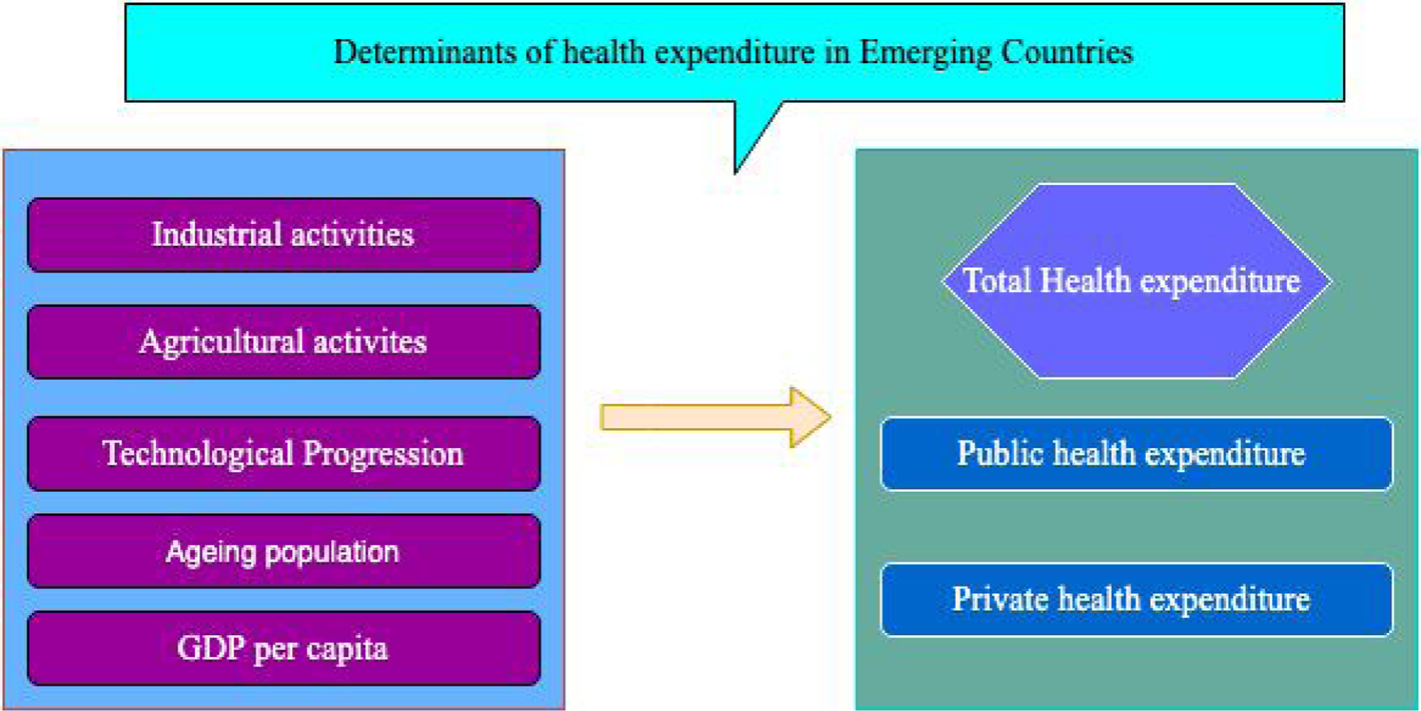
Factors influencing health expenditure in emerging economies

Figure 2 provides an overview of the different factors that influence health expenditures in emerging nations. Bergstra, Brunekreef [19], Mudu, Terracini [13], Hawkesworth, Dangour [20] Ejigu and Mekonennem [21] and [22] investigated the impact of industrial and agricultural activities on health outcomes established the effect of these activities on health expenditure. An increase in the aging population has been confirmed by [23] to influence health expenses. Bedir [24] established that income levels stimulate healthcare expenditures. In studying the convergence and the influencing factors of healthcare costs among the OECD countries [25] found that the main driver of health expenditure is technological progress. Guided by the above literature, the study conceptualizes that industrialization, agricultural activities, technological advancement, 65 years and above aged populations, GDP per capita, affect private and public healthcare expenses. Below is the Conceptual framework of the study as seen in Fig. 2.

## Econometric method and data sources

### Data

The study used datasets encompassing twenty-two (22) sample emerging countries based on the Internal Monetary Fund’s and Morgan Stanley Capital classifications^7^: Egypt, Indonesia, India, Pakistan, Philippines, Bangladesh, Argentina, Brazil, Colombia, Peru, Thailand, China, Venezuela, South Africa, Czech Republic, Chile, Russian Federation, Poland, Ukraine, Bulgaria, and Malaysia. Romania, Nigeria, Korea, and Qatar were not included in the study due to the non-availability of data. The reason for studying the 22 Emerging nations is that these nations are related to each other in terms of many socioeconomic indicators including growth rate and development. Besides, the selection of the study countries is governed by the availability of data and the fact that the researcher’s keen interest is to assess the determinants of health expenditure among these nations in the new millennium. Datasets employed were extracted annually from the World Development Indicators starting from 2000 to 2018.^8^ The data comprises public healthcare expenditure, out-of-pocket payments, population with ages 65 years and above, GDP per capita, industrialization, agricultural activities, and technological advancement. Industrialization comprises value added in mining, manufacturing, construction, electricity, water, and gas. The definitions of the study variables are presented in Table 1.

**Table 1:** Variable Definition and Source

Acronym	Variable name	Unit	Source
PHCE	Public health care expenditure	Current US $	WDI (2019)
OOP	Out-of-pocket payment	Current US $	WDI (2019)
POP	Population with ages 65 and over	Individuals above 65 years old	WDI (2019)
TEC	Patent application	Residents and non-residents per capita	WDI (2019)
AGRIC	Agriculture, value added	current US$	WDI (2019)
INDUS	Industrialization	current US$	WDI (2019)
PGDP	Gross Domestic Product per capita	current US$	WDI (2019)

Table 2 describes the features of the data set employed in the study. The mean, median, maximum, minimum, standard deviation (SD) estimations were performed in the descriptive analysis. In considering the main panel, Table 2 revealed industrialization (INDUS) as the variable with the highest mean (25.281). Agriculture had the highest standard deviation of (18.816), indicating agricultural as a critical variable in the study. Industrialization again is found as the variable with the highest Maximum value of (36.079). However, the individual countries revealed Bangladesh as a nation with the lowers minimum value of (1.943), (2.093), and (6.0) for out-of-pocket payments, public health expenditure, and GDP per capita. In the case of technological advancement, Ukraine had the minimum value of (20.96) whiles Peru had (3.296) a minimum value for Agricultural activities. Venezuela and Indonesia also showed a minimum value of (13.865), (8.102) for population and industrialization. The following nations have the highest maximum values: Brazil (6.346) for out-of-pocket payment, Venezuela nation with the highest maximum value for technological advancement, agricultural activities, population, and industrialization respectively.

**Table 2:** Descriptive Statistics

		Mean	Std. Dev.	Min	Max
Panel	lnOOP	4.219	1.040	1.624	6.364
	lnPHCE	5.270	1.260	2.093	7.543
	lnGDP	8.282	0.978	7.024	10.042
	lnTEC	23.670	1.334	21.096	27.609
	lnAGRIC	7.576	1.984	3.296	14.219
	lnPOP	15.427	1.203	13.718	18.865
	lnINDUS	25.281	1.634	21.816	36.079
	lnOOP	4.10	0.36	3.53	4.58
	lnPHCE	4.58	0.38	3.97	5.05
Egypt	lnGDP	7.62	0.43	6.97	8.19
	lnTEC	23.81	0.41	23.14	24.39
	lnAGRIC	7.46	0.32	6.54	7.71
	lnPOP	15.21	0.13	15.03	15.45
	lnINDUS	24.80	0.58	24.03	25.53
	lnOOP	3.288	0.734	1.943	4.113
	lnPHCE	4.068	0.723	2.788	4.918
Indonesia	lnGDP	16.282	0.110	16.114	16.496
	lnTEC	26.106	0.633	24.961	26.749
	lnAGRIC	7.640	0.602	6.618	8.267
	lnPOP	24.967	0.618	23.968	25.618
	lnINDUS	8.629	0.362	8.102	9.201
	lnOOP	3.261	0.397	2.589	3.825
	lnPHCE	3.641	0.448	2.921	4.277
India	lnGDP	6.930	0.516	6.094	7.609
	lnTEC	26.095	0.505	25.334	26.750
	lnAGRIC	10.262	0.584	9.052	10.834
	lnPOP	17.938	0.175	17.655	18.241
	lnINDUS	26.593	0.596	25.573	27.32492
	lnOOP	2.843	0.403	2.131	3.363
	lnPHCE	3.248	0.368	2.641	3.775
	lnGDP	6.816	0.387	6.181	7.295
Pakistan	lnTEC	24.312	0.538	23.478	24.980
	lnAGRIC	6.985	0.278	6.428	7.460
	lnPOP	15.844	0.156	15.577	16.072
	lnINDUS	24.220	0.440	23.500	24.763
	lnOOP	3.623	0.681	2.533	4.430
	lnPHCE	4.261	0.597	3.308	5.011
	lnGDP	7.503	0.441	6.864	8.040
Philippines	lnTEC	23.716	0.443	23.033	24.197
	lnAGRIC	7.979	0.336	6.750	8.225
	lnPOP	15.109	0.235	14.748	15.473
	lnINDUS	24.716	0.491	23.993	25.346
	lnOOP	2.459	0.587	1.624	3.384
	lnPHCE	2.860	0.535	2.093	3.694
	lnGDP	6.601	0.477	6.024	7.437
Bangladesh	lnTEC	23.631	0.395	23.145	24.302
	lnAGRIC	5.759	0.065	5.666	5.869
	lnPOP	15.109	0.235	14.748	15.473
	lnINDUS	24.002	0.621	23.199	25.083
	lnOOP	4.973	0.323	4.201	5.324
	lnPHCE	6.516	0.534	5.409	7.169
	lnGDP	9.023	0.511	7.861	9.588
Argentina	lnTEC	23.823	0.446	23.023	24.337
	lnAGRIC	8.471	0.177	8.095	8.800
	lnPOP	15.109	0.235	14.748	15.473
	lnINDUS	25.186	0.459	24.120	25.669
	lnOOP	5.533	0.726	4.486	6.364
	lnPHCE	6.290	0.711	5.357	7.171
	lnGDP	8.852	0.531	7.948	9.491
Brazil	lnTEC	24.863	0.502	24.014	25.456
	lnAGRIC	10.038	0.233	9.706	10.338
	lnPOP	16.364	0.226	15.996	16.738
	lnINDUS	26.407	0.542	25.462	27.127
	lnOOP	3.87	0.73	2.38	4.58
	lnPHCE	5.59	0.49	4.84	6.15
	lnGDP	8.46	0.46	7.73	9.01
Colombia	lnTEC	23.38	0.38	22.79	23.76
	lnAGRIC	7.45	0.38	6.21	7.77
	lnPOP	14.78	0.23	14.45	15.19
	lnINDUS	24.86	0.55	24.01	25.55
	lnOOP	4.217	0.467	3.485	4.769
	lnPHCE	5.274	0.493	4.502	5.900
Peru	lnGDP	8.306	0.489	7.571	8.846
	lnTEC	22.833	0.504	22.136	23.442
	lnAGRIC	4.182	1.067	3.296	6.983
	lnPOP	14.374	0.173	14.072	14.668
	lnINDUS	24.373	0.573	23.433	24.983
	lnOOP	3.163	0.146	2.914	3.339
	lnPHCE	4.923	0.504	4.048	5.544
	lnGDP	8.308	0.447	7.546	8.892
Thailand	lnTEC	22.833	0.504	22.136	23.442
	lnAGRIC	6.822	0.258	6.280	7.360
	lnPOP	15.577	0.208	15.230	15.921
	lnINDUS	25.343	0.468	24.504	25.897
	lnOOP	4.235	0.646	3.237	5.155
	lnPHCE	5.010	0.875	3.746	6.228
	lnGDP	8.139	0.813	6.866	9.187
China	lnTEC	26.855	0.639	25.904	27.609
	lnAGRIC	12.343	1.336	10.140	14.219
	lnPOP	18.535	0.168	18.284	18.865
	lnINDUS	28.340	0.811	27.036	29.342
	lnOOP	5.390	0.610	4.425	6.303
	lnPHCE	6.342	0.804	5.131	7.543
Venezuela	lnGDP	9.146	0.609	8.093	10.010
	lnTEC	23.196	0.810	21.997	24.321
	lnAGRIC	4.221	0.302	3.555	4.796
	lnPOP	14.215	0.207	13.865	14.491
	lnINDUS	27.528	4.437	22.433	26.079
	lnOOP	5.592	0.416	4.926	6.146
	lnPHCE	6.574	0.510	5.782	7.216
South Africa	lnGDP	9.196	0.466	8.400	9.676
	lnTEC	22.596	0.432	21.852	23.109
	lnAGRIC	5.890	0.199	5.485	6.275
	lnPOP	14.242	0.1932544	13.952	14.578
	lnINDUS	24.737	0.508	23.828	25.216
	lnOOP	4.90	0.65	3.55	5.46
	lnPHCE	6.93	0.49	5.84	7.35
Chile	lnGDP	9.63	0.42	8.70	10.04
	lnTEC	21.97	0.33	21.38	22.36
	lnAGRIC	6.59	0.19	6.27	6.89
	lnPOP	14.30	0.13	14.16	14.54
	lnINDUS	24.70	0.43	23.76	25.11
	lnOOP	4.863	0.730	3.361	5.644
	lnPHCE	5.930	0.672	4.558	6.698
Czech Republic	lnGDP	8.881	0.707	7.480	9.681
	lnTEC	24.457	0.498	23.427	25.008
	lnAGRIC	7.827	1.635	6.265	9.750
	lnPOP	16.778	0.036	16.719	16.866
	lnINDUS	26.478	0.681	25.202	27.197
	lnOOP	5.03	0.37	4.30	5.38
	lnPHCE	6.40	0.46	5.47	6.82
Russia	lnGDP	9.20	0.42	8.41	9.64
	lnTEC	23.03	0.35	22.39	23.44
	lnAGRIC	8.42	0.33	7.92	8.95
	lnPOP	15.48	0.10	15.34	15.70
	lnINDUS	25.41	0.43	24.63	25.80
	lnOOP	4.1	0.6	2.8	4.9
	lnPHCE	4.9	0.6	3.6	5.7
Poland	lnGDP	7.7	0.6	6.5	8.3
	lnTEC	22.9	0.4	22.2	23.5
	lnAGRIC	8.5	0.3	7.9	9.1
	lnPOP	15.8	0.0	15.7	15.8
	lnINDUS	24.0	0.5	23.0	24.7
	lnOOP	5.073	0.693	3.642	5.891
	lnPHCE	5.900	0.629	4.549	6.611
Ukraine	lnGDP	8.534	0.566	7.384	9.135
	lnTEC	21.491	0.197	21.096	21.886
	lnAGRIC	5.869	0.566	5.355	6.974
	lnPOP	14.133	0.039	14.100	14.208
	lnINDUS	22.948	0.5123	21.321	23.721
Bulgaria	lnOOP	4.231	0.712	3.212	5.110
	lnPHCE	5.032	0.621	4.212	6.612
	lnGDP	8.432	0.654	7.210	9.21
	lnTEC	24.032	0.212	23.321	25.022
	lnAGRIC	7.123	1.432	6.108	9.212
	lnPOP	16.032	0.0321	16.198	16.312
	lnINDUS	26.212	0.654	25.431	27.212
	lnOOP	4.48	0.47	3.72	5.04
	lnPHCE	5.50	0.48	4.71	6.04
	lnGDP	8.91	0.39	8.27	9.33
Malaysia	lnTEC	23.63	0.51	22.73	24.25
	lnAGRIC	8.68	0.27	7.77	8.95
	lnPOP	14.12	0.25	13.72	14.54
	lnINDUS	25.17	0.41	24.48	25.65

### The procedures involved in the analysis of the study

**Fig. 3:**
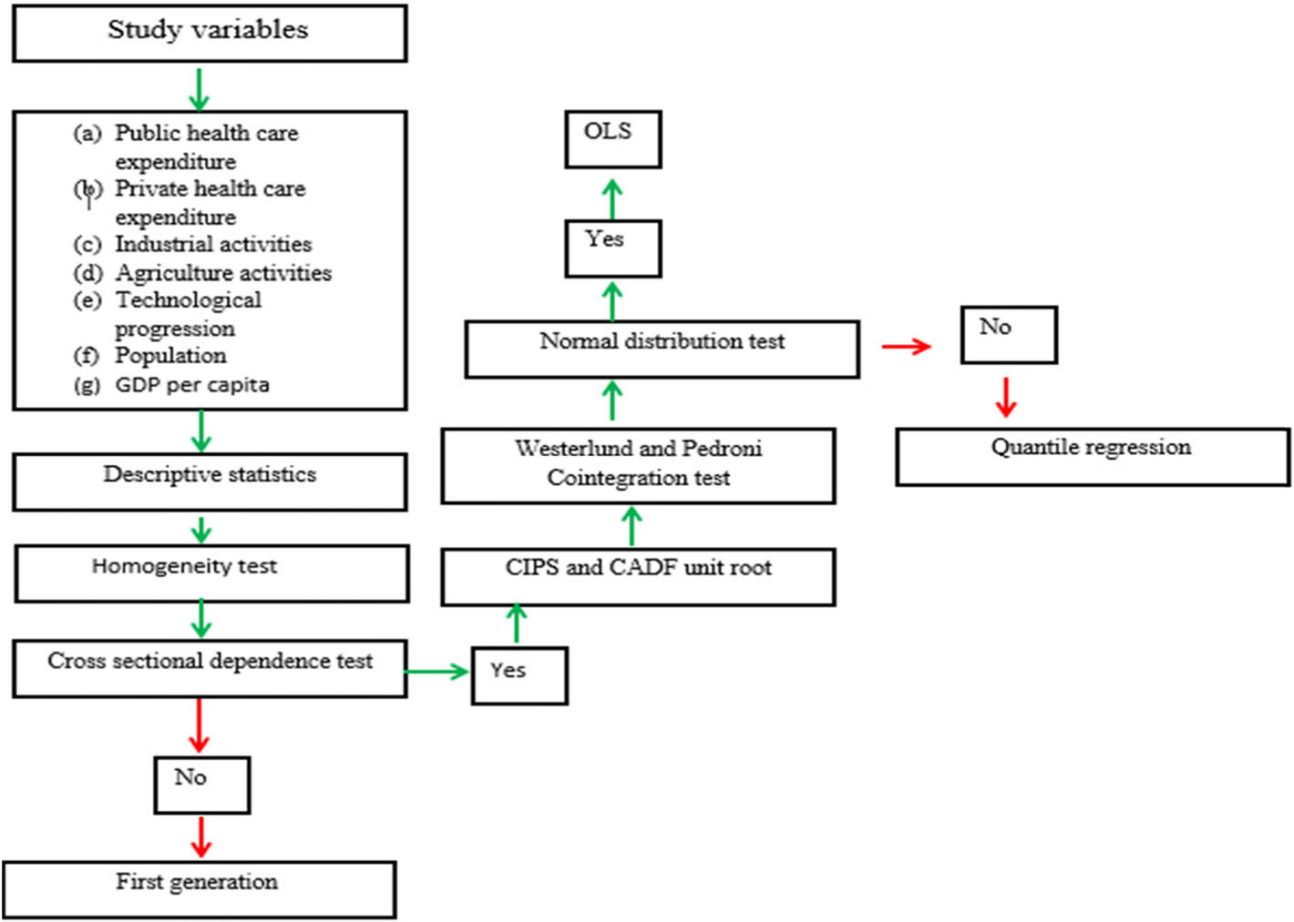
Schematic steps of the study

The methods employed in the study are shown in Fig. 3. To begin with, the descriptive statistic is performed to summarize the characteristics of the data set employed in the study. It is succeeded by the homogeneity test, this test aims to enable the researchers to know whether the gradient coefficients are homogeneous or not. The cross-sectional dependent test is performed to help the researchers examine the most common issue in the panel country-level data set, which is the interdependence of the individual countries. Hence ignoring the cross-sectional dependent test can result in invalid results. This assisted the researchers to choose the appropriate unit root procedures. Based on the outcome of the cross-sectional dependent and the homogeneity test, the first generation unit root technique is invalid for the study as it relies on cross-section dependence in data series. Hence the second generation unit root (panel cross-sectional augmented (CIPS) and cross-sectional-augmented Dicker-Fuller (CADF) unit root test) is applied to determine the order of integration of the variables. The Westerlund and Pedroni cointergration is used to examine the long-run relationship between the variables. Following the confirmation of the long-run relationship among the variables the Pooled mean group method to check the causal connections among the variables. The normality test is performed and the coefficient of kurtosis showed that the variables are not normally distributed. Based on the findings of the quantile regression method the estimation of the long run connections between the study variables is conducted.

### Panel Quantile regression model

The quantile regression test proposed by Koenker and Bassett [20], is employed to eliminate the limitations of the ordinary least square (OLS) technique. The standard form panel quantile regression is expressed as eq. 1

1 $$ {Quant}_0{y}_i/{x}_i={x\beta}_0+{\mu}_0,0 b\theta b1 $$

where y represents the endogenous variables while x indicates the exogenous variables, whereas *μ* shows the error term in the *θ* th distribution point of the endogenous variables. The theoretical background of this study was founded on the endogenous growth model Romer [21] of which the projected output (health expenditure) depends on industrialization, economic growth, agricultural production, the population including technological advancement. The study transformed the variables into natural log to adjust for the skewed distribution of the datasets. The model is the therefore presented as;

2 $$ {lnPHCE}_{it}={\alpha}_{it}+{\omega}_1{lnAGRIC}_{it}+{\omega}_2{lnPGDP}_{it}+{\omega}_3{lnINDUS}_{it}+{\omega}_4{lnPOP}_{it}+{\omega}_5{lnTEC}_{it}+{\varepsilon}_{it} $$

3 $$ {lnOOP}_{it}={\alpha}_{it}+{\omega}_1{lnAGRIC}_{it}+{\omega}_2{lnPGDP}_{it}+{\omega}_3{lnINDUS}_{it}+{\omega}_4{lnPOP}_{it}+{\omega}_5{lnTEC}_{it}+{\varepsilon}_{it} $$

here AGRIC is agricultural activities, PGDP is a gross domestic product, POP denote population, INDUS represents industrialization, TEC is technological progression. Quantile regression of panel is employed to estimate the influence of the regressors on public and private healthcare expenditure at the designated quantile levels; hence follows eq. 4 and 5;

4 $$ {Q}_{\tau}\left({lnPHCE}_{it}\right)={\alpha}_{\tau }+{\omega}_{1\tau }{lnAGRIC}_{it}+{\omega}_{2\tau }{lnPGDP}_{it}+{\omega}_{3\tau }{lnINDUS}_{it}+{\omega}_{4\tau }{lnPOP}_{it}+{\omega}_{5\tau }{lnTECNO}_{it}+{\xi}_I $$

5 $$ {Q}_{\tau}\left({lnOOP}_{it}\right)={\alpha}_{\tau }+{\omega}_{1\tau }{lnAGRIC}_{it}+{\omega}_{2\tau } lnPGD{\mathrm{P}}_{it}+{\omega}_{3\tau }{lnINDUS}_{it}+{\omega}_{4\tau }{lnPOP}_{it}+{\omega}_{5\tau }{lnTECNO}_{it}+{\xi}_I $$

here *Q*_*τ*_ indicates regression parameters for *τth* distributional point and *τ* represents the distribution point of the exogenous variables.

### Homogeneity and cross-sectional dependence tests

In the course of identifying stationary characteristics of healthcare financing, out-of-pocket payment, income, population, industrialization, and technological advancement require elaboration on the features of the panel data. This will enable the study to employ a suitable panel unit root test to prevent ambiguous results. The study first tested for homogeneity using Pesaran and Yamagata [26] test and the outcome is shown in Table 3. The findings in Table 3 disclosed the need for denial of the null homogeneity statement of the coefficients and therefore acceptance of the alternative statement of heterogeneity. Following the homogeneity test, the study scrutinized cross dependence between the variables utilizing Cross-sectional dependence (CD) test [27]. Pesaran gives a robust means of dealing with spillover effects because the countries have comparable features. Precisely, the null statement assumes cross-sectional independence within the series while the alternative hypothesis indicates cross-sectional dependence.

**Table 3:** Results of Homogeneity Test

PHCE			OOP	
Test	statistics	*p*-value	statistics	*p*-value
Delta_tilde	299.6	0	263.5	0.007**
Delta_tilde _adj	5.302	0	−0.941	0.346

**Statistically significant at 5% level of significance

Table 4 below provides the findings of the CD test. Based on the results of the cross-sectional dependence check, the denial of the null assumption and acknowledgment of the alternative statement that, there exists a cross-sectional dependence within data series was indispensable.

**Table 4:** Results of Cross-sectional dependence

Variables	CD-test	*p*-value
lnPHCE	55.4	0.000***
lnOOP	51.14	0.000***
lnPOP	54.71	0.000***
lnAGRIC	56.9	0.000***
lnIND	54.84	0.000***
lnGDP	57.4	0.000***
lnTEC	53.1	0.000***

***Statistically significant at 1% level of significance

Before analyzing the long-run association between PHCE and the determinants, the study first observes collective stationarity property of the variables. The reason is that estimating variables with unit root dilemma is found to be spurious and detrimental for policymaking. Therefore, there is a need for an examination of the stationarity features of the study variables. Since the findings of the cross-sectional-dependence test indicate the presence of cross-sections among the variables, the first generation unit root technique became invalid for the study as it relies on cross-section dependence in data series. On that account, the panel cross-sectional augmented (CIPS) and cross-sectional-augmented Dicker-Fuller (CADF) unit root test were performed [28]. CIPS and CADF unit root test is implemented to determine the order of integration of each variable.

The findings in Table 5 prove that nearly all the variables provide evidence for the existence of unit root among data series except technological advancement, agricultural production, and income. Consequently, all the variables are stationary in the first difference.

**Table 5:** Results unit root test results

CIPS test					CADF test			
	Levels		*∆*		Levels		*∆*	
Variables	Statistics	*P*-value	Statistics	*P*-value	Statistics	*P*-value	Statistics	*P*-value
lnPHCE	− 2.002	0.624	−3.923	0.005**	−1.412	0.929	−2.986	0.000***
lnOOP	− 2.16	0.432	−3.782	0.000***	−1.701	0.569	− 2.799	0.000***
lnPOP	−2.662	0.546	−2.137	0.005**	−1.651	0.656	−2.799	0.008**
lnAGRIC	−3.392	0.052*	−4.198	0.001***	−2.282	0.007**	−2.391	0.020**
lnIND	−1.369	0.876	−2.835	0.031**	−1.304	0.975	−2.372	0.023**
lnGDP	−2.614	0.065*	−3.504	0.002***	−1.718	0.539	−2.366	0.002***
lnTec	−2.522	0.021**	−4.114	0.001***	−2.455	0.001***	−2.274	0.008**

∆ denotes first difference, *,**,** and * represent 10, 5 and 1% level of significances respectively

After the panel unit root determination, the Westerlund cointegration test was applied to examine the long-run linkages among the study variables [29]. Westerlund, Edgerton panel cointegration was considered to deal with any cross-sectional dependence. The Westerlund cointegration statistics provide robustness of G_a_, G_t_, P_a_, and P_t_: G_a_ (among groups); G_t_ (between groups); P_a_ (among panels), and P_t_ is the robustness between panels. Evident in Table 6 is proof of cointegration; the existence of a long-run relationship between the variables. In addition, the robust p values support the rejection of the null statement of no cointegration and acceptance of the alternative hypothesis an indication of a long-run relationship between the series. On these grounds, there is a panel indication of a long-run connection within study variables.

**Table 6:** Westerlund cointegration test

Statistic	Value	Z-value	*P*-value	Robust *P*-value
G_t_	−3.059	−2.842	0.002	0.084 *
G_a_	−4.465	5.160	1.000	0.033**
P_t_	−4.014	4.798	1.000	0.675
P_a_	−0.701	4.871	1.000	0.838

*, **, represent 10, and 5% level of significances respectively

The Pedroni cointegration test results shown in Table 7 below is the cointegration values of the variables. Estimation from Panel rho-Statistic, Panel v-Statistic, and Group rho-Statistic was insignificant of Pedroni cointegration leading to the acceptance of the null assumption of no cointegration within the data series. However, Panel ADF-Statistic, Panel ADF-Statistic, Group PP-Statistic, and Group ADF-Statistic shows significant values. Subsequently, the Panel ADF-Statistic, Group PP-Statistic, Panel ADF-Statistic, and Group ADF-Statistic rejected the null statement at a 5% significant level and therefore the alternative statement of cointegration in the data series was accepted. This also confirms the Westerlund test of cointegration showing long-run connections within the series.

**Table 7:** Pedroni cointegration test results

Alternative hypothesis: common AR coefs. (within-dimension)					
				Weighted	
		Statistic	Prob.	Statistic	Prob.
Panel v-Statistic		1.562221	0.0591*	− 0.83359	0.7977
Panel rho-Statistic		2.756464	0.9971	1.200641	0.8851
Panel PP-Statistic		−0.25363	0.3999	−6.48389	0.000***
Panel ADF-Statistic		−3.87437	0.0001***	−4.13868	0.000***
Alternative hypothesis: individual AR coefs. (between-dimension)					
		Statistic	Prob.		
Group rho-Statistic		3.144622	0.9992		
Group PP-Statistic		−8.34968	0.000***		
Group ADF-Statistic		−3.16744	0.000***		

*and, *** represent 10 and 1% level of significances respectively

Following the Westerlund cointegration test, Pedroni [30] cointegration was again performed to verify the long-run association between the data series. As demonstrated in Table 7, the null theory stating cointegration is non-existing according to Pedroni test is rejected, leading to the acceptance of the alternative theory at the 5% significance level. Before continuing with the panel quantile regression method, it is necessary to check the normal distribution trend within the data. This study, therefore, adopted the Shapiro-Francia Royston [31] and Shapiro-Wilk Royston and computing [32] normality checks for assessing normal distribution within the data.

Table 8 presents the results revealed by the Shapiro-Francia test and Shapiro-Wilk tests. On the grounds of the statistical output, the outcome rejects the null statement at a 1% significance level, a sign that the data is not normally scattered. Kurtosis and Skewness tests were again performed to confirm the normal distribution to the study variables. Whereas skewness p.values beginning with 0, is an indication that the values are not normally distributed, the value of Kurtosis measures the level of the flatness of the series. The recorded coefficient of kurtosis is greater than 3, an indication that the variables are not normally distributed. Therefore the quantile regression is considered valid in cases where the study variables are found not to be normally distributed.

**Table 8:** Normal distribution test

Variables	Obs.	Skewness	Kurtosis	Shapiro-Wilk test		Shapiro-Francia test	
				Statistics	Sig.	Statistics	Sig.
**lnPHCE**	399	1.547	4.984	0.973	0.000***	0.975	0.000***
**lnIndus**	399	10.310	110.356	0.788	0.000***	0.785	0.000***
**lnoop**	399	1.849	6.514	0.987	0.002***	0.989	0.007**
**lnpop**	399	3.236	13.144	0.901	0.000***	0.903	0.000***
**lngdp**	399	1.161	3.8280	0.970	0.000***	0.973	0.000***
**lnagri**	399	4.579	25.798	0.961	0.000***	0.963	0.000***
**lntech**	399	7.798	67.820	0.976	0.000***	0.977	0.000

*** signify at 1 percent level of significance

**Fig. 4:**
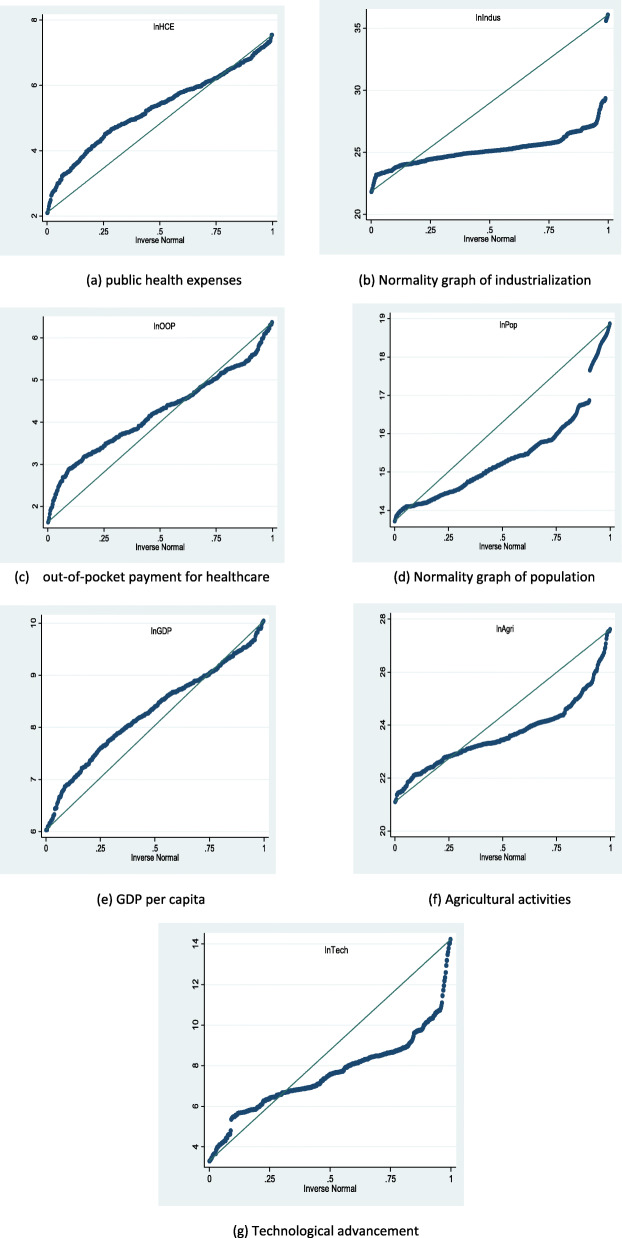
**a**-**g** Normal distribution graph of public healthcare expenditure, industrialization, out-of-pocket payment for healthcare, aging population, GDP per capita, agricultural activities and technological progression

Figure 4a-g, depict a graphical presentation of the study variables distributions. Distinctively, the blue line shows the predictable normal distribution. However, as observed from Fig. 4a-g, public healthcare expenditure and out-of-pocket expenditure did not fall on the normally distributed line. Similarly, the regressors including agriculture activities, per capita GDP, technological progression, population, and industrialization again failed to be distributed normally. Consequently, the traditional ordinary least squares (OLS) regression could have estimation biases, since OLS can be considered valid in cases where the study variables are found to be normally distributed. For this reason, the panel regression method was employed to overcome the shortcomings of conventional OLS. This study adopts panel quantile regression.

Figure 4a-g depicts a graphical presentation of the study variables’ normal distributions. Distinctively, the blue line shows the predictable normal distribution. As observed from Fig. 4a-g, public healthcare expenditure and out-of-pocket expenditure did not fall on the normally distributed line. Similarly, the regressors including agriculture activities, per capita GDP, technological progression, population, and industrialization again failed to be distributed normally. Consequently, the traditional ordinary least squares (OLS) regression could have estimation biases, since OLS can be considered valid in cases where the study variables are found to be normally distributed. For this reason, the panel quantile regression method was employed to overcome the shortcomings of conventional OLS.

After performing the preliminary procedures, the study proceeded with the quantile regression procedures to consider the factors influencing the trend of health expenditure within the emerging nations. Two models were developed to find the disaggregated effects of the regressors on public and private healthcare financing (out-of-pocket payments). To this end, quantile panel regression offers a graphical explanation of the marginal impact of regressors on the endogenous variables. Nine (9) quantile levels were selected namely the 15th, 25th, 35th, 45th, 55th, 65th, 75th, 85th, and again 95th) to attain a comprehensive analysis.

Table 9 reports the outcomes of the impacts of income, industrialization, economic growth, technological innovation, and agricultural activities on public health financing. Given this, the OLS estimation method was considered for the goal of comparison. Industrialization has both statistically positive and negative coefficients denoting that industrialization could cause an increase or reduction in public healthcare expenditure. Specifically, a unit increase in industrialization will reduce health expenses at the 15th and 25th quantile by (0.208), and (0.319) units but will increase public health financing at the 35th, 65th, 75th, 85th and 95th quantile by (0.519), (0.049), (0.476) and (0.427) units.

**Table 9:** Results of quantile regression procedure Public health expenditure

Variable	OLS	Quantiles								
		15th	25th	35th	45th	55th	65th	75th	85th	95th
**lnIND**	0.022 (0141)	−0.208* (0.111)	−0.136 (0.128)	0.319* (0.141)	0.0476 (0.8262)	0.053 (0.561)	0.591*** (0.025)	0.049** (0.212)	0.476** (0.189)	0.427*** (0.072)
**lnPOP**	0.110*** (0.262)	0.273*** (0.484)	0.277*** (0.562)	0.488*** (0.086)	0.0919* (0.5937)	0.113** (0.413)	0.118*** (0.029)	0.104 (0.046)	0.5842 (0.462	0.560* (0.294)
**lnGDP**	1.237*** (0.190)	1.395*** (0.632)	1.337*** (0.793)	1.233*** (0.911)	1.199*** (0.0538)	0.113*** (0.037)	1.219*** (0.038)	1.261*** (0.039)	1.264*** (0.037)	1.229*** (0.033)
**lnAGRI**	−0.201** (0.243)	0.975* (0.574)	− 0.171** (0.773)	0.199* (0.113)	0.2293* (0.9871)	−0.285*** (0.071)	− 0.29*** (0.146)	0.276*** (0.428)	− 0.27*** (0.042)	− 0.18*** (0.254)
**lnTEC**	0.3423*** (0.115)	0.0775* (0.277)	0.001 (0.177)	0.004 (0.0213)	0.0387 (0.115)	0.0502*** (0.011)	0.064*** (0.442)	0.0824*** (0.150)	0.1.01*** (0.013)	0.095*** (0.123)
**R**^**2**^	0.953	0.807	0.804	0.793	0.792	0.794	0.792	0.791	0.789	0.787
**Durbin-Watson stat**	**2.016959**									

Standard deviations are in parenthesis. ***, **, *represents 1, 5 and10% significance level

Moreover, agricultural activities were positive and significant at all quantile levels. GDP per capita was positive and significant at all the quantile levels too. Thus a unit increase in income per capita can potentially upsurge public health expenditure by (1.237), (1.395), (1.337), (1.233) (1.199.), (0.113, (1.219), (1.261), (1.264.), (1.22) and (1.22) units respectively at the various quantile levels. These findings suggest that income per capita economic growth is one of the main factors affecting public health spending in emerging countries. Technological advancement was positive and significant at all quantile levels except the 25th, 35th^,^ and 45th quantile levels an indication that technological advancement contributes to an increase in public health expenses too.

Table 10 present the results of the effects of income, industrialization, economic growth, technological innovation, and agricultural activities on out-of-pocket payments. Categorically, a unit increase in Industrialization was found to be statistically negative at 15th (0.022) and 25th (0.136), and OLS by (0.46) units. Moreover, a surge in the aging population increases out-of-pocket payment by (0.192) at the 45th quantile whereas OLS remained insignificant. The aging population only shows a positive and significant increase at the 45th quantile level. The GDP per capita revealed a positive and significant at all the quantile levels. Thus a unit increase in income per capita will increase out-of-pocket payments by (1.119), (1.130.), (1.055), (1.130), (0.874), (0.893), (0.985), (1.008) and (1.122) units, respectively. Agricultural activities revealed mixed results. A positive and significant relationship at the 15th (0.397), 25th (0.253), and the 35th (0.193) units quantile level and a negative and significant relationship (0.140) was revealed at the 85th quantile level. This result recommends that agriculture activities either could induce or reduce out-of-pocket payment in emerging economies. Technological advancement demonstrated a positive and significant association at the 15th quantile level suggesting that technological advancement contributes to an increase in out-of-pocket payment. To detect whether the model has a serial correlation issue, the Durbin-Watson test is employed. The Durbin Watson (DW) statistic results from Table 10 showed positive autocorrelation whiles findings from Table 9 revealed no autocorrelation among the study variable.

**Table 10:** Results of quantile regression procedure of Out-of-pocket payments

Variable	OLS	Quantiles								
		15th	25th	35th	45th	55th	65th	75th	85th	95th
lnIND	−0.460* (0.262)	−0.522** (0.164)	− 0.307 (0.231)	− 0.206 (0.239)	−0.131 (0.255)	0.059 (0.286)	0.399 (0.233)	0.0217 (0.123)	0.1306 (0.062)	−.012 0.086
lnPOP	0.002 (0.048)	0.106 (0.093)	0.185 (0.116)	0.152 (0.150)	0.192** (0.101)	0.124 (0.117)	0.010 (0.121)	0.469 (0.870)	0.844 (0.074)	0.114 (0.128)
lnGDP	0.976** (0.035)	1.149*** (0.087)	1.130*** (0.133)	1.055*** (0.140)	1.031*** (0.153)	0.874*** (0.187)	0.893*** (0.162)	0.9850*** (0.095)	1.008*** (0.0639)	1.124*** 0.508
lnAGRI	0.125 (0.450)	0.397*** (0.053)	0.253*** (0.797)	0.193** (0.096)	0.050 (0.134)	0.105 (0.187)	−0.801 (0.190)	−0.118 (0.118)	−0.140** (0.067)	0.106 (0.109)
lnTEC	0.011*** (0.021)	0.963** (0.062)	0.0277 (0.675)	−0.034 (0.306)	−0.028 (0.224)	0.018 (0.020)	0.0362 (0.304)	0.3914 (0.445)	0.029 (0.0304)	0.0584 (0.044)
R^2^	0.763	0.546	0.549	0.555	0.553	0.540	0.548	0.562	0.565	0.588
**Durbin-Watson stat**	1.98									

Standard deviations is in parenthesis. ***, **, *represents 1, 5 and10% significance level

To aid in the formulation of appropriate policies, decision-makers must know causal relationships between endogenous and exogenous variables. Given this, Pooled Mean Group (PMG) suggested by Pesaran, Shin [33] was estimated. Table 11 presents the findings of the pooled mean causality tests showing unidirectional causality run between industrialization and public health expenses; public health spending and population; out-of-pocket payment and technological advancement; out-of-pocket payments and agricultural activities.

**Table 11:** Pooled Mean Group (PMG) Causality test

Variable	Coefficients	t- statistics	Probability	Conclusions
INDUS→ PHEC	0.019 (0.025)	0.789	0.430	Unidirectional causality between PPHCE and INDUS
PHCE →INDUS	0.347 (0.039)	−8.735	0.000***	
PHCE →POP	0.549 (0.109)	5.426	0.000**	Unidirectional causality between PHCE and POP
POP → PHCE	0.018 (0.062)	0.789	0.432	
PHCE →GDP	0.495 (0.296)	16.704	0.000***	Bidirectional causality between PHCE and PGDP
PGDP → PHCE	0.743 (0.053)	14.013	0.000***	
PHEC →TEC	0.979 (0.076)	12.763	0.000***	Bidirectional causality between PHCE and TEC
TEC → PHEC	−0.020 (0.009)	−2.149	0.032**	
PHEC → AGRIC	0.322 (0.054)	5.879	0.000**	Bidirectional causality between PHCE and AGRIC
AGRIC → PHCE	2.729 (0.046)	5.983	0.000***	
OOP → INDUS	−0.109 (0.072)	−6.334	0.000***	Bidirectional causality between OOP and INDUS
INDUS→ OOP	−0.090 (0.048)	−1.861	0.064*	
OOP → POP	0.532 (0.202)	2.630	0.009**	Bidirectional causality between OOP and POP
POP →OOP	−0.629 (0.214)	−2.929	0.003***	
OOP → PGDP	0.025 (0.014)	13.740	0.000***	Bidirectional causality between OOP and PGDP
PGDP → OOP	1.068 (0.118)	9.035	0.000***	
TEC → OOP	−0.035 (0.022)	−1.573	0.117	Unidirectional causality between OOP and TEC
OOP → TEC	0.171 (0.055)	3.084	0.002v**	
OOP → AGRIC	0.1549 (0.051)	3.095	0.002***	Unidirectional causality between OOP and AGRIC
AGRIC →OOP	−0.123 (0.110)	−1.112	0.267	

Standard deviation is in parenthesi s. ***, **, * represents 1, 5 and 10% significance level, respectively

The empirical findings, however, revealed a bidirectional causality between public health spending and GDP per capita; public health financing and technological advancement; public health financing and agricultural activities; out-of-pocket payment, and industrialization; out-of-pocket payments and aging population; and out-of-pocket payments and GDP per capita.

## Discussion

In seeking explanations to the determinants of health expenses in the emerging economies, the study considered certain contributing factors. The study revealed that industrialization could potentially reduce or increase public health spending. The implications of these results are twofold. On one hand, increased industrial activities have been identified as a significant source of emission causing environmental carcinogens and other non-communicable diseases [34]. The health consequences from these industrial activities may compel governments to increase spending to improve the health status of affected individuals. On the other hand, as emerging nations experience a surge in industrialization, there are massive employment opportunities that can cause a spike in GDP per capita. An increase in employment as a result of industrialization may escalate household income and decreases economic hardship, both of which can improve physical and psychological well-being and reducing the demands on the public health care system.

The study again revealed that agricultural activities could increase public health spending. This can be explained by the fact that although most emerging countries are currently undergoing a major transition in agriculture by embracing new technologies including agrochemicals and mechanization to secure food for the masses. The introduction of these new types of agricultural machinery and agrochemicals has been confirmed to have adverse public health such as serious injuries, and cancer that could subsequently lead to health consequences hence increasing government health expenses. On the other hand, agriculture activities could decrease public healthcare expenditure when it aims at producing foods that are nutritious to boost immunity to fight diseases. When this happens, it decreases the occurrences of nutritional related diseases and the government may not need to hugely invest in the healthcare system. The study again indicated that economic growth positively affects both public health care expenses at all levels. The study is in support of the argument that, as countries’ income increases, they tend to spend more on population health. This is indicative that, as the economy grows, more health goods such as multifaceted health equipment are purchased by the governments to assist in effective and efficient healthcare delivery. This acknowledges the findings of [25, 35]. It, however, departs from Han, Cho’s [6] longitudinal study on the factors influencing healthcare expenses among sixteen (16) provinces in Korea from the year 2003–2010 in which a conclusion of no significant relationship between income and health expenditure was reported.

Another deduction from the study is that an upsurge of the aging population within emerging economies will upturn public and private health expenses. This could be attributed to the fact that the elderly population (with ages sixty-five (65) and above) could have deterioration of health status leading to chronic diseases or disabilities. The findings of the study coincide with similar findings from Byaro, Kinyondo [36]; Khan, Razali [4] who concluded that an increase of public health expenses could correlate with a surge of the aging population. An indication that the aging population is a significant contributor in explaining the increase in health care expenses.

Technological advancement increases public health financing at all quantile levels. The results of our study suggest that advances in technology have a significant role in health care expenditure within emerging nations. This means that although with an advance in technology, some diagnostic tests and drugs have become much cheaper, its effects on healthcare expenditure within the emerging nations have not been fully realized. The positive relationship between technology and health expenditure suggests that the use of sophisticated items such as diagnostic imaging systems, organ transplantation, and new treatment modalities for treaments can be very expensive. Banz and Eucomed [37] emphasized particular areas of technology advancement that has been accompanied by more spending, including revascularization for coronary artery disease and joint replacement. The study, therefore, encourages the need for further research to be geared towards investigating the effects of medical technology on health outcomes within the selected study countries. Similar conclusions from [4, 38] postulate that as nations move towards technological advancements, diseases previously ignored because of lack of treatments are now recognized and managed as a result of technological progression and this too comes at an expensive cost.

Industrialization, on the other hand, showed a negative relationship with out-of-pocket payment for healthcare. A potential explanation of this finding is that an increase in industries that invests in environmental protection measures can minimize health hazards and contribute effectively to maintaining health. For instance, most industrial companies give health insurance for workers and affected community members to minimize financial barriers to access to health care a key role in reducing health inequalities. This conclusion is supported by [39] assessment based on a comprehensive review of the literature.

The study again revealed that agricultural activities could be a double-edged sword by reducing or increasing private health spending. This implies that agricultural activities may provide cheaper and less variable micronutrients, foods, and plants with medicinal properties and increase productivity, which will generate more income to enable individuals to finance health care. In the other direction, agricultural activities are associated with other health problems. As a result workers with poor health are less able to work, generate income and this in turn can perpetuate a downward spiral into ill health and poverty, with an increased burden indirect cost in accessing healthcare.

Concerning private health expenses, a surge in income levels has been identified to increase private healthcare expenditure. One potential explanation for this result is that the poor mostly delay and avoid seeking healthcare services due to the inability to afford health costs. Others such as [40] in Kenya, [41] for Albania, and [42] in Nigeria found that poorer individuals and households experience lower out-of-pocket expenditures on health care than wealthier households.

Further, the study, on the one hand, identified unidirectional causality existing between industrialization and public health expenses; public health spending and population; out-of-pocket payment, and technological advancement; and out-of-pocket payments and agricultural activities. The unidirectional causality between the variables indicates that the one-directional influence of a variable resulting in a high-level effect of the other variable exists such that the other variable does not affect the influencing variable. On the other hand, a two-way causality amongst public health expenditure and GDP per capita; public health expenditure and technological advancement; public health expenditure and agricultural activities; out-of-pocket payments and industrialization; out-of-pocket payments and aging population; and out-of-pocket payments and GDP per capita was revealed. A bidirectional Pooled Mean Group causality between the variables indicates that the above variables determined jointly and also affect each other at the same time.

## Conclusion

The study provides an empirical analysis of the determinants of health expenditure in the selected emerging economies. The study data extracted from the World Development Bank Indicators (WDI) covers the period of 2000 to 2018. Econometric procedures known to be robust and providing better statistical interpretations in the existence of heterogeneity and spatial dependence were employed for data analysis. The findings show that the availability of heterogeneous and cross-sectional dependent variables that require the use of second-generation methods. The study, therefore, employed the CADF and CIPS unit root tests to deal with heterogeneity and cross-sectional dependence. The findings of the Westerlund and Pedroni cointegration tests substantiated a long-run equilibrium linkage between the study variables.

Additionally, the study employed the quantile regression method to subdue the estimation bias of the ordinary least square approach in the case where there is no normal distribution between the variables. The results revealed that economic growth and the aging population (with ages 65 years and above) induces health costs in the emerging countries. However, the impact of industrialization, agricultural activities, and technological advancement on health expenses are found to be noticeably heterogeneous at the various quantile levels.

### Managerial implication of the study

Universal health coverage embodies national health systems in which all individuals can access quality health services without individual or familial financial hardship. However, insufficient funds for health could impede the expansion of universal health coverage. Most challenging, perhaps, is the need to increase health financing rapidly enough to facilitate universal coverage of essential health services among emerging nations. The interactions between agriculture activities and out-of-pocket payment for health care are two-way; thus a positive and negative relationship. Other abatement strategies should be implemented to reduce the role of agricultural activities in increasing out-of-pocket expenditure within the emerging nations. The study reveals that an increase in the elderly population contributes to increasing health costs in emerging economies. The study, therefore, recommends health policymakers to design/modify and intensify health educational programs seeking to improve the health and prevention of diseases among the aging population. The findings showed a mixed effect of certain variables on the upsurge of health spending in the countries under investigation. The factors include industrialization, agricultural activities, and technological advancement. In light of this finding, it is obvious that a new concept for cleaner and safer agricultural practices that will encourage a drastic reduction in the use of chemical pesticides is required to promote consumers’ health. Findings from the causality tests indicate that enhancement of the health expenses should be a major priority during policy development in the emerging countries since investments and expansions in the health sector play a significant role in the attainment of other developmental processes.

This study is not without its limitations. One limitation of the study is that it did not consider other emerging economies and variables like corruption in the analysis due to the unavailability of data, which will be considered in our forthcoming studies. In subsequent studies, it is worthwhile for an expanded scope to explore the influence of governance on healthcare spending in the emerging economies to better understand the role of governance indicators, and how it influences healthcare expenses in these countries.

## Data Availability

The data used to support the findings of this study was extracted from World Bank World Development Indicator WDI (2018) under license and copyright of World Bank World Development Indicators so it cannot be made freely available by authors. Requests for access to these data should be made to World Bank World Development Indicator WDI (2018) http://datatopics.worldbank.org/world-development-indicators/.

## Abbreviations

WHO: World Health Organization
OOP: Out of Pocket Payments
US$: US dollars
OLS: Ordinary Least Square
ARDL: Autoregressive Distributed Lag Model
OECD: Organization for Economic Cooperation and Development
SD: Standard Deviation
CV: Coefficient of Variations
INDUS: Industrialization
CD test: Cross-sectional dependence test
CIPS: Cross-sectional augmented CIPS
CADF: Cross-sectional-augmented Dicker-Fuller
Ga: Among groups
Gt: Between groups
Pa: Among panels
PHCE: Public health care expenditure
POP: Population with ages 65 and over
TEC: Technological innovation
AGRIC: Agricultural activity
PGDP: Gross Domestic Product per capita

## Footnotes

1 Global spending on health is expected to increase to $18.28 trillion worldwide by 2040 but many countries will miss important health benchmarks

2 World Health Organization (WHO) / World Bank. 2017. *Tracking Universal Health Coverage: 2017 Global Monitoring Report*.

3 https://www.ubs.com/content/dam/WealthManagementAmericas/documents/emerging-market-healthcare.pdf

4 https://www.ubs.com/content/dam/WealthManagementAmericas/documents/emerging-market-healthcare.pdf

5 The Global Economic Burden of Non-Communicable Diseases. September 2011. World Economic Forum and Harvard School of Public Health.

6 https://www.genevaassociation.org/sites/default/files/research-topics-document-type/pdf_public/health_protection_gap_web.pdf

7 https://www.thebalance.com/what-are-emerging-markets-3305927.

8 http://datatopics.worldbank.org/world-development-indicators.
